# Inhibition of Autophagy by Deguelin Sensitizes Pancreatic Cancer Cells to Doxorubicin

**DOI:** 10.3390/ijms18020370

**Published:** 2017-02-10

**Authors:** Xiao Dong Xu, Yan Zhao, Min Zhang, Rui Zhi He, Xiu Hui Shi, Xing Jun Guo, Cheng Jian Shi, Feng Peng, Min Wang, Min Shen, Xin Wang, Xu Li, Ren Yi Qin

**Affiliations:** Department of Biliary-Pancreatic Surgery, Affiliated Tongji Hospital, Tongji Medical College, Huazhong University of Science and Technology, Wuhan 430030, China; tjxuxiaodong@163.com (X.D.X.); zhaoy2401@163.com (Y.Z.); zm0911work@163.com (M.Z.); heruizhi@aliyun.com (R.Z.H.); 18771146878@163.com (X.H.S.); amjoyguo@126.com (X.J.G.); chengj1010@sina.com (C.J.S.); nicholas.peng7@gmail.com (F.P.); wangmin0013128@aliyun.com (M.W.); shenming-2002@163.com (M.S.); xwangtjh@163.com (X.W.)

**Keywords:** pancreatic cancer, deguelin, autophagy, doxorubicin

## Abstract

Pancreatic cancer is the fourth most common cause of cancer mortality worldwide. Furthermore, patients with pancreatic cancer experience limited benefit from current chemotherapeutic approaches because of drug resistance. Therefore, an effective therapeutic strategy for patients with pancreatic cancer is urgently required. Deguelin is a natural chemopreventive drug that exerts potent antiproliferative activity in solid tumors by inducing cell death. However, the molecular mechanisms underlying this activity have not been fully elucidated. Here we show that deguelin blocks autophagy and induces apoptosis in pancreatic cancer cells in vitro. Autophagy induced by doxorubicin plays a protective role in pancreatic cancer cells, and suppressing autophagy by chloroquine or silencing autophagy protein 5 enhanced doxorubicin-induced cell death. Similarly, inhibition of autophagy by deguelin also chemosensitized pancreatic cancer cell lines to doxorubicin. These findings suggest that deguelin has potent anticancer effects against pancreatic cancer and potentiates the anti-cancer effects of doxorubicin. These findings provide evidence that combined treatment with deguelin and doxorubicin represents an effective strategy for treating pancreatic cancer.

## 1. Introduction

Pancreatic ductal adenocarcinoma (PDAC) is the fourth leading cause of cancer-related death in the United States of America [1]. The lack of effective treatments, especially for those with advanced disease, leads to a poor prognosis for PDAC patients. Therefore, new and more effective therapeutic options for PDAC are required, and combination therapies are being developed [2,3].

Autophagy is a conserved cellular degradation process whereby cellular organelles and proteins are engulfed by autophagosomes, digested within lysosomes, and recycled to maintain cellular homeostasis [4,5,6]. In cancer, autophagy plays important roles both in cell death and survival [7,8]. Autophagy is activated in response to a number of stressors, including cancer chemotherapeutics, facilitating cell survival and leading to treatment resistance. Meanwhile, certain conditions and stimuli promote overactivation of autophagy, which ultimately causes cell death. Therefore, the function of autophagy in cell death and survival during cancer treatment requires consideration.

Autophagy is constitutively activated in pancreatic cancer, and this autophagy is required for tumor growth both in vivo and in vitro [9]. Inhibition of autophagy promotes tumor regression and increased survival in a mouse model of PDAC, indicating that enhanced autophagy is essential for PDAC tumorigenesis and/or growth [10]. Additionally, combination treatment with autophagy inhibitors such as chloroquine (CQ) potentiates the effects of several anti-cancer therapeutic agents [11,12]. Therefore, inhibition of autophagy may be useful in sensitizing pancreatic cancer cells to chemotherapeutic treatment.

Deguelin, a retinoid extracted from *Mundulea sericea* (Willd), exerts pro-apoptotic activity in various cancer models including those of breast, gastric, and prostate cancer [13,14,15]. Previous studies have shown that deguelin induces apoptosis in cancer cells by targeting phosphoinositide-3 kinase (PI3K)/Akt pathway [16]. Furthermore, deguelin inhibits the growth and metastasis of pancreatic cancer cells both in vivo and in vitro [17,18]. However, the mechanism by which deguelin modulates autophagy has not been thoroughly investigated. In this work, we demonstrate that deguelin induces apoptosis and inhibits autophagy in pancreatic cancer cell lines. Furthermore, deguelin-mediated inhibition of autophagy chemosensitizes pancreatic cancer cell lines to doxorubicin. These results suggest that deguelin may be an effective agent to use in combination with doxorubicin, and that such a combination therapy could serve as a novel strategy in treating pancreatic cancer.

## 2. Results

### 2.1. Deguelin Inhibits Pancreatic Cancer Cell Growth and Induces Apoptosis

**Figure 1:**
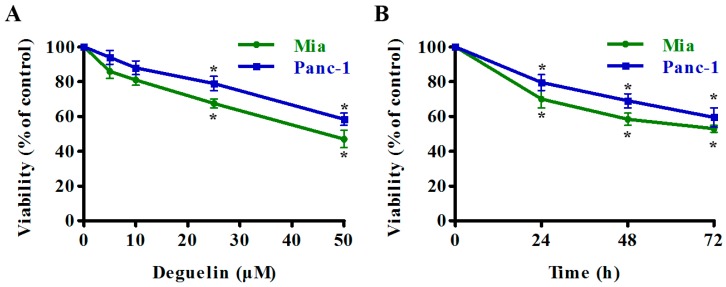

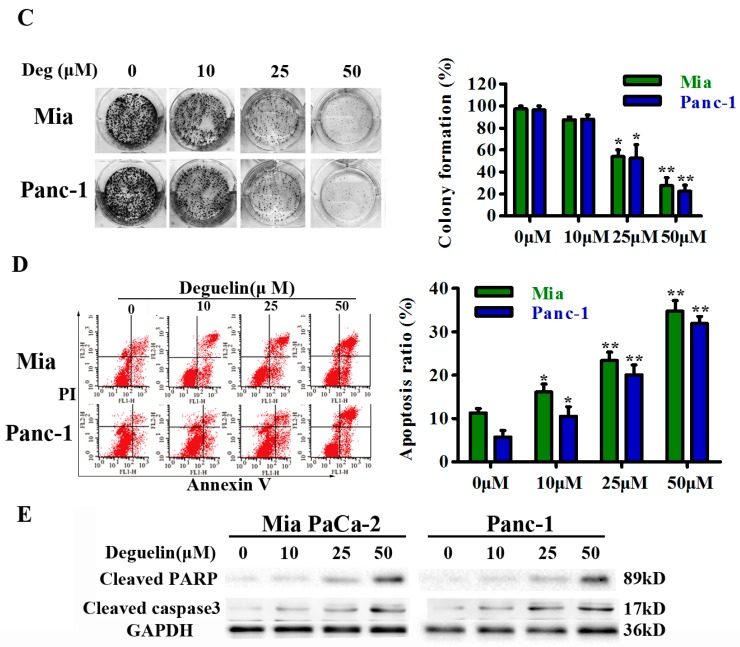
Deguelin inhibits pancreatic cancer cell growth and induces apoptosis. (**A**,**B**) Mia PaCa-2 and Panc-1 cells were treated with deguelin at indicated concentrations or time points. Cell Counting Kit-8 assay was performed to evaluate cell proliferation; (**C**) Representative images from the clonogenic assays. Mia PaCa-2 and Panc-1 cells were incubated with indicated concentrations of deguelin and cultured for 14 days. Each bar represents means ± Standard Deviation (SD) of three separate experiments (right); (**D**) Significant increase in apoptosis after cells were treated as described in (**C**). Apoptotic cells (Annexin V^+^) were analyzed and results are presented as the mean ± SD (right); (**E**) Western blot analysis for cleaved PARP and cleaved caspase-3 was performed on lysates from cells treated as described in (**C**). GAPDH from a similarly loaded gel is shown as loading control. * *p* < 0.05, ** *p* < 0.01 versus the control group. GAPDH: glycerinaldehyde-3-phosphat-dehydrogenase.

We determined the effect of deguelin on pancreatic cancer cells by treating two pancreatic cancer cell lines Mia PaCa-2 and Panc-1 cells with increasing concentrations of deguelin for 24 h, or with 25 μM deguelin for different time periods. Cell viability was assessed by CCK-8 cell proliferation and cytotoxicity assays. Deguelin partially inhibited pancreatic cancer cell growth, and deguelin-mediated cytotoxicity in these cells was both dose- and time-dependent (Figure 1A,B). Next, we used a clonogenic assay to demonstrate that deguelin affected long-term colony formation by markedly inhibiting the number of surviving colonies (Figure 1C). We then investigated whether deguelin induced apoptotic cell death using the Fluorescein Isothiocyanate (FITC)-labeled Annexin V/PI staining and flow cytometry. Pancreatic cancer cell lines treated with deguelin underwent apoptosis within 24 h of treatment in a dose-dependent manner (Figure 1D). Consistently, deguelin induced a dose-dependent increase in the levels of cleaved caspase-3 and cleaved PARP—one of the cellular substrates of caspases-3 (Figure 1E). Taken together, these results indicate that deguelin inhibits proliferation and induces apoptosis of human pancreatic cancer cell lines.

### 2.2. Deguelin Induces Incomplete Autophagy in Pancreatic Cancer Cells

**Figure 2:**
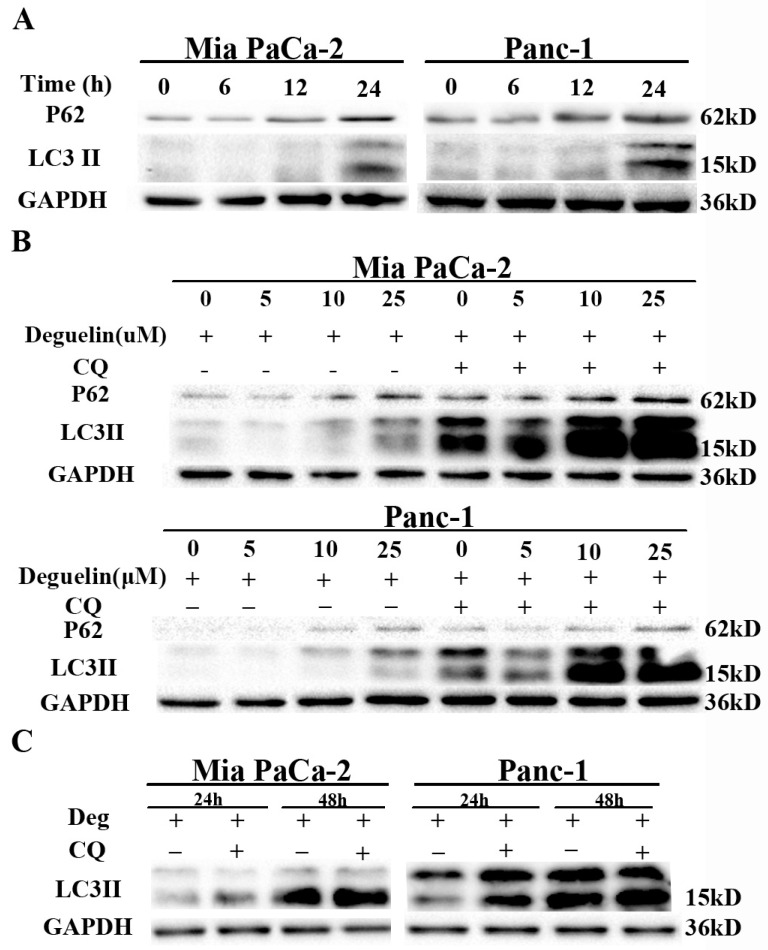
Deguelin induces incomplete autophagy in pancreatic cancer cells. (**A**) Mia PaCa-2 and Panc-1 cells were treated with 25 μM of deguelin for the indicated time points. Protein expression levels of LC3-II and p62 were measured by western blot; (**B**) Mia PaCa-2 and Panc-1 cells were treated with deguelin at the indicated concentrations for 24 h, with or without chloroquine (CQ). Cell lysates were analyzed by western blot; (**C**) Mia PaCa-2 and Panc-1 cells were treated with deguelin (25 μM) and/or CQ (10 μM) for 24 or 48 h and cell lysates were analyzed by western blot. GAPDH from a similarly loaded gel is shown as loading control.

Earlier studies have shown that deguelin possesses anti-tumor activity, but the mechanism of action is unclear [18,19]. We first monitored autophagic alterations by analyzing the abundance of autophagosome marker LC3-II and the multifunctional cargo protein p62/SQSTM1 after deguelin treatment. Treatment with deguelin induced accumulation of LC3-II and impaired p62 clearance in both dose- and time-dependent manners (Figure 2A,B). Increased numbers of autophagosomes may be associated either with increased autophagosome synthesis or decreased autophagosome maturation and degradation. However, deguelin induced an increase in levels of the multifunctional cargo protein p62, indicating that deguelin inhibited autophagy flux. Additionally, treatment of cells with CQ—which blocks late stage autophagy by impairing lysosomal acidification—promoted ac cumulation of higher levels of LC3-II and p62 when compared with deguelin treatment alone (Figure 2B). These findings suggest that deguelin induced incomplete autophagy in these cells. Accordingly, expression levels of the early stage autophagy-related proteins Beclin1, Atg3, and Atg5 were also investigated. In both cell lines, deguelin markedly increased levels of Beclin1, Atg3, and Atg5 (Figure S1). Additionally, treatment of cells with 50 μM deguelin for 48 h with or without CQ yielded no difference in LC3-II levels (Figure 2C), indicating that a high concentration of deguelin saturated the ability of CQ to block autophagic flux.

**Figure 3:**
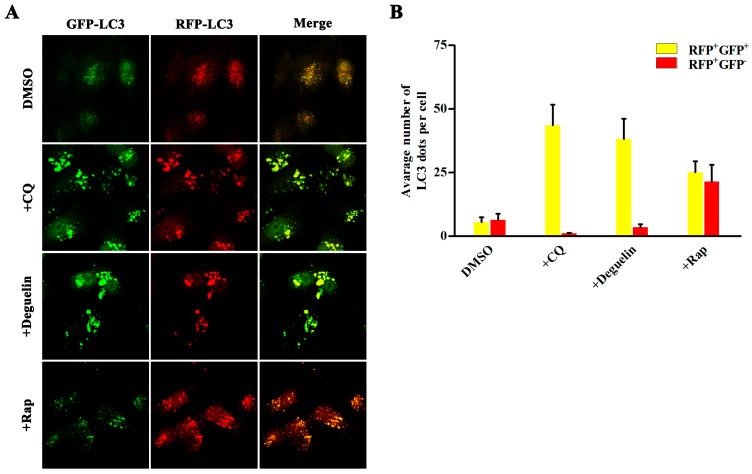
Deguelin inhibits autophagosome maturation. (**A**) Mia PaCa-2 cells were transfected with GFP-mRFP-LC3B for 48 h and then treated with vehicle DMSO, Chloroquine (10 μM), deguelin (25 μM), or Rapamycin (100 nM) for 12 h, and green and red fluorescence was detected using a confocal microscope. Right panel, the numbers of acidified autophagosomes (GFP^−^RFP^+^) versus neutral autophagosomes (GFP^+^RFP^+^) per cell in each condition were quantified; (**B**) Data are presented as the means ± SD from three independent experiments.

We further investigated whether deguelin suppresses the progression of autophagy using a tandem-labeled GFP-mRFP-LC3 construct, which is a useful tool to examine autophagosome maturation and autolysosome formation. In this assay, mRFP is more resistant to pH changes, whereas GFP is sensitive to pH and is quenched in the acidic environment of the lysosome. Therefore, the fusion of autophagosomes with lysosomes results in the loss of yellow puncta and the appearance of red-only puncta. Figure 3A demonstrates that treatment of cells with rapamycin induces a yellow color in some of the LC3B-positive puncta. Contrastingly, treatment with either CQ or deguelin promoted formation of both GFP and mRFP puncta that extensively co-localized with each other and thus appeared yellow. These findings suggest that deguelin blocked autophagosome maturation in a similar fashion to CQ.

### 2.3. Doxorubicin Induces Autophagy in Pancreatic Cancer Cells

**Figure 4:**
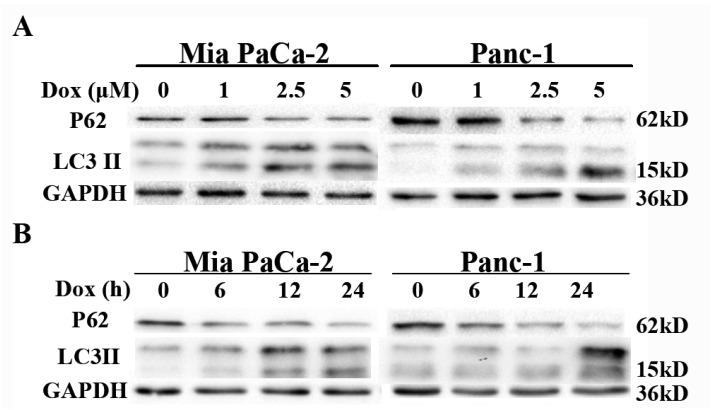

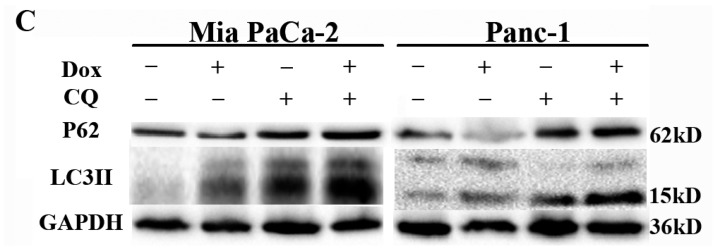
Doxorubicin induces autophagy in pancreatic cancer cells. (**A**,**B**) Doxorubicin induces autophagy in pancreatic cancer cells. Western blot analysis of LC3-II and p62 protein levels in Mia PaCa-2 and Panc-1 cell lines treated with indicated concentrations of doxorubicin for 24 h, or treated with 2.5 μM doxorubicin for indicated times; (**C**) LC3-II turnover assay during doxorubicin-induced autophagy. Mia PaCa-2 and Panc-1 cells were treated with CQ (10 μM) and/or doxorubicin (2.5 μM) for 24 h, and cell lysates were collected and subjected to western blot.

Doxorubicin is a DNA damage-inducing first-line antineoplastic drug used in the treatment of various cancers [20]. We next investigated whether doxorubicin induces autophagy in pancreatic cancer using cultured Mia PaCa-2 and Panc-1 cells. Treatment with doxorubicin induced aggregation of LC3-II and decreased levels of p62 in dose- and time-dependent manners in both cell lines (Figure 4A,B). Moreover, treatment with CQ enhanced the levels of LC3-II in cells also treated with doxorubicin (Figure 4C). These findings indicate that doxorubicin induced increased autophagic flux.

### 2.4. Autophagy Protects Pancreatic Cancer Cells from Doxorubicin-Induced Cell Death

**Figure 5:**
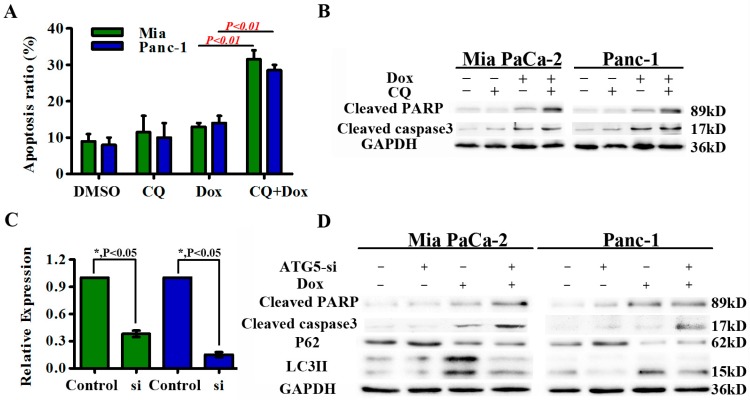

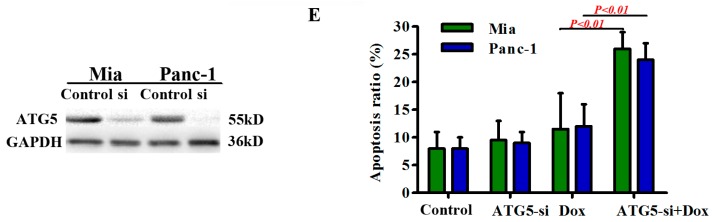
Autophagy has a pro-survival role in doxorubicin-induced cell death. (**A**,**B**) Mia PaCa-2 and Panc-1 cells were treated with doxorubicin (2.5 μM) in the presence or absence of CQ (10 μM). The percentage of Annexin V positive cells was recorded. Cleaved PARP and cleaved caspase-3 levels were also analyzed by western blot; (**C**) Mia PaCa-2 and Panc-1 cells were transfected with siRNA against the essential autophagy gene Atg5 or with a scrambled siRNA. Atg5 mRNA and protein expression levels were detected by real-time quantitative PCR and western blot, respectively. Results are presented as the means ± SD; (**D**) Western blot analysis of autophagic markers (LC3-II and p62) and apoptotic markers (cleaved PARP and cleaved caspase-3) from Mia PaCa-2 and Panc-1 cells transfected with the indicated siRNAs followed by doxorubicin treatment for 24 h; (**E**) Evaluation of apoptosis in Mia PaCa-2 and Panc-1 cells following suppression of autophagy by knockdown of Atg5 and treatment with doxorubicin (2.5 μM) for 24 h. GAPDH from a similarly loaded gel is shown as loading control.

The role of autophagy in induction of cell death depends on the context and stimulus. We next assessed apoptosis in cells treated with doxorubicin in the presence or absence of CQ treatment. Combined treatment with CQ significantly enhanced doxorubicin-induced apoptosis (Figure 5A). This increased apoptosis was further confirmed by increased levels of cleaved PARP and cleaved caspase-3 (Figure 5B).

CQ may impact other cellular processes in addition to autophagy, so we next blocked autophagy by silencing expression of Atg5, a protein essential for autophagosome expansion and completion. Small interfering RNA-mediated knock-down of Atg5 expression in Mia PaCa-2 and Panc-1 cells was confirmed by PCR and western blot (Figure 5C). Silencing of Atg5 significantly reduced autophagic flux in doxorubicin-treated cells as indicated by decreased LC3-II and increased p62 levels determined by western blot (Figure 5D). Atg5 knock-down in Mia PaCa-2 and Panc-1 cells augmented doxorubicin-induced cell death, indicating a pro-survival role for autophagy in doxorubicin-induced cell death (Figure 5E). Levels of cleaved-PARP and cleaved caspase-3 were also increased (Figure 5D). These findings indicate that autophagy induced by doxorubicin treatment of pancreatic cancer cells serves as a pro-survival mechanism, while inhibition of autophagy enhances the anticancer effects of doxorubicin.

### 2.5. Deguelin Enhances the Cytotoxic Effects of Doxorubicin by Suppressing Autophagic Flux

**Figure 6:**
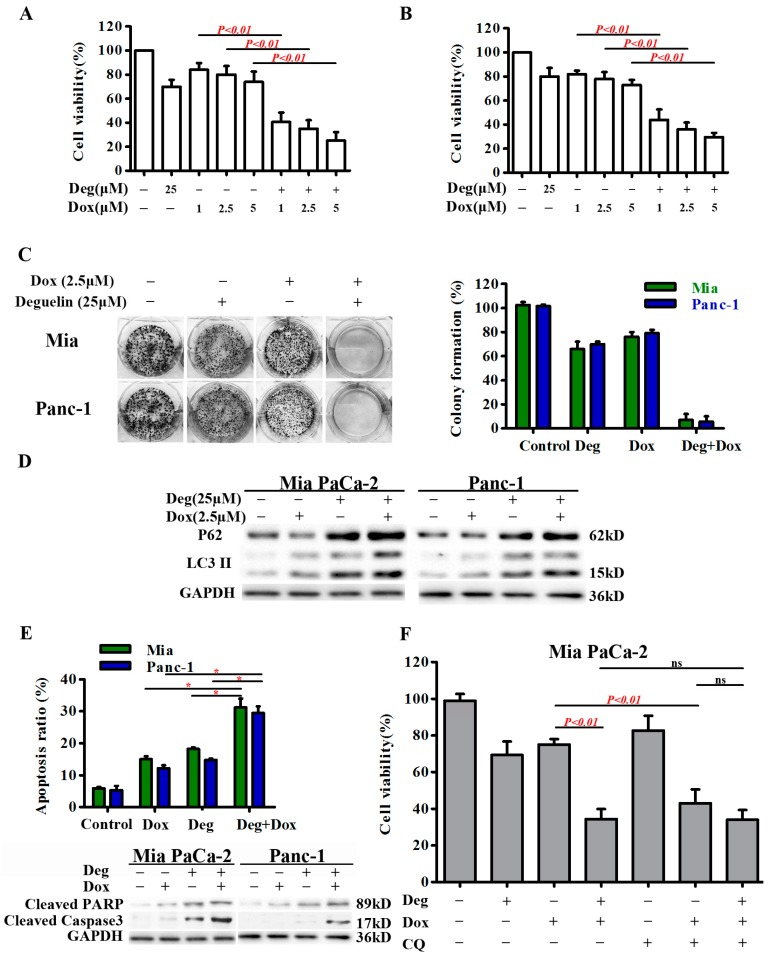

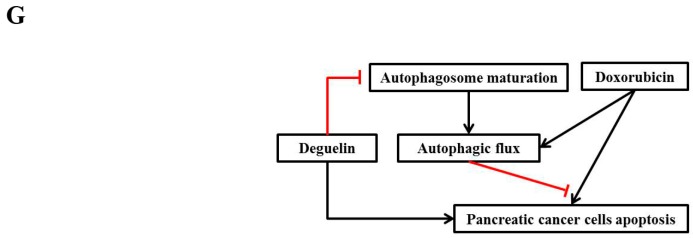
Deguelin impairs autophagy and potentiates doxorubicin-induced cytotoxicity in pancreatic cancer cells. (**A**,**B**) Mia PaCa-2 and Panc-1 cells were incubated with increasing doses of doxorubicin (1–5 μM) and deguelin (25 μM) for 24 h. Cell proliferation was then determined by CCK-8 assay; (**C**) Mia PaCa-2 and Panc-1 cells were treated with 2.5 µM doxorubicin alone or in combination with 25 µM deguelin for 2 weeks with growth media changed every 2–3 days. Cell proliferation was analyzed using a colony formation assay; (**D**) LC3-II turnover assay in Mia PaCa-2 and Panc-1 cells treated with 25 µM deguelin and 2.5 μM doxorubicin for 24 h alone or in combination. Cell lysates were collected and subjected to western blot for LC3-II, p62 and GAPDH protein expression; (**E**) Mia PaCa-2 and Panc-1 cells were treated with 2.5 µM doxorubicin alone or in combination with 25 µM deguelin for 24 h; * *p* < 0.05. Cell apoptosis was quantified by Annexin V-FITC/PI double staining. The results are presented as the means ± SD from at least three independent experiments; Cleaved PARP and cleaved caspase-3 levels were also analyzed by western blot; (**F**) Mia PaCa-2 and Panc-1 cells were treated with 2.5 µM doxorubicin alone or in combination with 25 µM deguelin or 10 µM CQ for 24 h. Cell viability was an analyzed by CCK-8 assay. Results are presented as the means ± SD. GAPDH from a similarly loaded gel is shown as loading control; (**G**) This schematic model depicts that deguelin reduces doxorubicin-induced autophagy and sensitizes pancreatic cancer cells to doxorubicin.

We next examined whether inhibition of autophagy by deguelin could sensitize pancreatic cancer cells to doxorubicin-induced cell death using a CCK-8 assay. Mia PaCa-2 cells exposed to deguelin were significantly sensitized to doxorubicin-induced cell death (Figure 6A). Similar results were also obtained for Panc-1 cells (Figure 6B). However, no sensitization was observed following treatment of normal human pancreatic ductal epithelial (HPDE) cells with doxorubicin and deguelin (Figure S2). This suggests that deguelin synergism with doxorubicin is selective to cancer cells.

We next performed colony formation assays in which Mia PaCa-2 and Panc-1 cells were treated with doxorubicin (2.5 µM) and low concentrations of deguelin (25 µM), either alone or in combination. Mia PaCa-2 cells exhibited a 24% decrease in colony formation after exposure of otherwise untreated cells to doxorubicin (Figure 6C). Combination treatment with doxorubicin (2.5 µM) and deguelin (25 µM) induced a 93% decrease in colony formation compared with no treatment. In Panc-1 cells, there was a 21% decrease in colony formation after doxorubicin (2.5 µM) treatment compared with untreated cells (Figure 6C), while combination treatment with doxorubicin (2.5 µM) and deguelin (25 µM) induced a 94% decrease in colony formation compared with the control group. Deguelin enhanced doxorubicin-induced LC3-II accumulation and decreased the clearance of p62 (Figure 6D) in a manner similar to CQ, indicating that deguelin blocked doxorubicin-induced autophagic flux at a late stage. Mia PaCa-2 and Panc-1 cells treated with the combination of doxorubicin (2.5 µM) and deguelin (25 µM) underwent dramatic sensitization to doxorubicin-induced cell death (Figure 6E). While CQ and deguelin could each sensitize cells to doxorubicin on their own, combined treatment with CQ and deguelin had no additional sensitizing effect on doxorubicin-induced toxicity compared with deguelin alone (Figure 6F and Figure S3). These findings indicate that CQ and deguelin sensitized pancreatic cells to doxorubicin largely though the same mechanism. Taken together, these results suggest that autophagy functions as a survival mechanism during doxorubicin treatment, and that deguelin sensitized pancreatic cancer cells to doxorubicin-mediated cytotoxicity at least in part by suppressing autophagy.

## 3. Discussion

Previous studies indicate that inhibition of autophagy enhances the efficacy of anticancer drugs, suggesting that suppression of autophagy may be an effective anti-cancer therapeutic strategy [21,22,23]. Currently, CQ and its derivative, hydroxychloroquine, are the only Food and Drug Administration-approved drugs that have been evaluated by phase I/II clinical trials across a range of tumor types [24]. Other compounds can also effectively inhibit autophagy, and deguelin may be one such compound with the potential to be developed into a novel anti-cancer therapeutic agent. Our current study demonstrates for the first time that deguelin, a rotenoid derived from *Mundulea sericea* (Willd) [15,16] with a chemical structure distinct from CQ, is a novel late-stage autophagy inhibitor. However, the effects of deguelin on autophagy in pancreatic cancer cells have not been previously reported.

We found that deguelin inhibits in vitro pancreatic cancer cell growth and induces apoptosis. Consistently, a recent study showed that deguelin could inhibit cancer cells growth with apoptosis being involved [17,25,26]. Furthermore, we found that deguelin promotes accumulation of autophagosomes. Autophagosome accumulation is an intermediate event within the autophagic flux process, reflecting the balance between the rate of autophagosome generation and degradation. Three possible mechanisms underlie this process: (1) deguelin induces complete autophagy; (2) deguelin simply suppresses basic autophagic flux; or (3) deguelin induces incomplete autophagy [27]. We used the lysosomal inhibitor CQ to further investigate these possibilities. Treatment with CQ dramatically increased the accumulation of LC3-II and p62 following deguelin treatment of both Panc-1 and Mia PaCa-2 cells, suggesting that deguelin induces incomplete autophagy in pancreatic cancer.

We further investigated the role of autophagy induced by doxorubicin, a common anti-cancer therapeutic agent that induces DNA damage. Although autophagy can mediate apoptotic cell death, the role of autophagy in cancer is contentious. Emerging evidence suggests that autophagy can promote cancer cell survival by maintaining energy production and is a critical mechanism of therapeutic resistance [16]. Here we demonstrate that doxorubicin induces protective autophagy in pancreatic cancer cells. Consistently, another report has described doxorubicin induced autophagy during induction of cell death [8]. Furthermore, pharmacologic or genetic inhibition of autophagy by knockdown of Beclin-1 or Atg5 expression augments doxorubicin-induced cell death in multiple myeloma cell lines [28].

Previous studies have shown that pancreatic cancer cells exhibit high expression levels of autophagy genes and proteins [9]. Furthermore, inhibition of active autophagy inhibits growth and enhances the sensitivity of pancreatic cancer cells to chemotherapy [2]. We confirmed that inhibition of autophagy using CQ, Atg5 siRNA, or deguelin induces increased cytotoxicity and apoptosis in pancreatic cancer cells.

Our results show that suppression of autophagy by deguelin markedly enhances doxorubicin-induced cell death in pancreatic carcinoma cells in vitro. Therefore, inhibition of autophagy could be an effective combination therapy to overcome chemoresistance and enhance chemotherapeutic efficacy.

## 4. Materials and Methods

### 4.1. Drugs and Reagents

Antibodies against human LC3-II, p62, Beclin1, Atg3, Atg5, Cleaved PARP, Cleaved caspase-3 and glycerinaldehyde-3-phosphat-dehydrogenase (GAPDH) were purchased from Cell Signaling Technology (Beverly, MA, USA). Doxorubicin, CQ, and deguelin were purchased from Sigma-Aldrich (St. Louis, MO, USA).

### 4.2. Cell Lines and Cell Culture

Human pancreatic cancer cell lines (Mia PaCa-2 and Panc-1) and the immortalized human pancreatic ductal epithelial cell line (HPDE) were purchased from the Cell Repository, Chinese Academy of Sciences (Shanghai, China). Mia PaCa-2 and Panc-1 cells were cultured in Dulbecco’s modified Eagle medium containing 10% inactivated fetal bovine serum (Gibico, Carlsbad, CA, USA), 1 × 10^5^ U/L penicillin, and 100 mg/L streptomycin (Gibico) in a humidified atmosphere with 5% CO_2_ at 37 °C. HPDE cells were grown in RPMI-1640 medium (Gibico) containing 10% inactivated fetal bovine serum (FBS). All cell lines used in this study were authenticated using short tandem repeat DNA profiling within six months and tested for mycoplasma contamination.

### 4.3. Cell Proliferation Assay

Cells were plated in 96-well culture plates (4 × 10^3^ cells per well) and treated with required compounds. After treatment, cell viability was measured by CCK8 assay (Dojindo Laboratories, Kumamoto, Japan) according to the manufacturer’s instructions. Three or four independent experiments were performed for each assay condition. The optical density values obtained for treatment wells were normalized to that of the control group.

### 4.4. Annexin V-FITC/PI Apoptosis Assay

Cell apoptosis was detected following specific treatments by staining using the Annexin V/PI apoptosis kit (MultiSciences, Hangzhou, China) according to the manufacturer’s instructions. Annexin V^+^ cells were detected using a flow cytometer (BD Immunocytometry Systems, San Jose, CA, USA). Each experiment was repeated three times.

### 4.5. Colony Formation Assay

Cells were plated at 500 cells per well in a 6-well plate. After 24 h, compounds were added as indicated in the figure legends. After rinsing with fresh medium, cells were incubated for 14 days. The medium was discarded and the cells were washed twice with PBS. After fixing cells with 4% paraformaldehyde for 15 min, cells were then stained with 1% crystal violet for 15 min. The number of colonies, defined as ≥50 cells/colony, was counted manually by light microscopy.

### 4.6. Western Blot Analysis

Cells subjected to desired treatments were lysed in RIPA buffer (Beyotime Biotechnology, Shanghai, China). Equal amounts of cell extracts were resolved on a 15% sodium dodecyl sulfide-polyacrylamide gel electrophoresis and transferred onto a polyvinylidene difluoride membrane (Millipore, Bedford, MA, USA). The blots were then incubated with relevant primary antibody overnight, followed by specific secondary antibodies coupled to horseradish peroxidase (1:2000, Boster, Wuhan, China), and visualized by enhanced chemiluminescence (Boster, Wuhan, China).

### 4.7. Real-Time PCR

Total RNA was extracted from pancreatic cancer cells using a TRIzol kit (Invitrogen, Carlsbad, CA, USA) according to the manufacturer’s instructions. RNA samples were run on an agarose gel to get electrophoretic profiles and detected by NanoDropND-2000 spectrophotometer (NanoDrop Tech., Wilmington, DE, USA) to detect the concentrations and values of A260/A280 and A260/A230, meanwhile the spectrum of each sample was also checked. cDNA was synthesized from 1 µg RNA using M-MLV Reverse Transcriptase (28025013; Invitrogen) according to the manufacturer’s instructions. The RT-qPCR assays of mRNA expression levels were performed using a SYBR Green PCR Kit (RR420A; Takara, Dalian, China) on ABI Prism 7500 (Applied Biosystems, Foster City, CA, USA) according to the manufacturer’s instructions. Housekeeping genes ACTB and glycerinaldehyde-3-phosphat-dehydrogenase (GAPDH) were used as reference genes. The validation of reference genes have been carried out according to minimum information for publication of quantitative real-time PCR experiments (MIQE) guidelines [29]. Comparable PCR efficiencies were calculated and checked by calibration curves. The primers used were: Atg5 forward: 5′-AAAGATGTGCTTCGAGATGTGT-3′, Atg5 reverse: 5′-CACTTTGTCAGTTACCAACGTCA-3′; GAPDH forward: AGAAGGCTGGGGCTCATTTG, GAPDH reverse: TGAGAGCTGTCCATTGGTAGAG; ACTB forward: TTGCCGACAGGATGCAGAAGGA, ACTB reverse: AGGTGGACAGCGAGGCCAGGAT. The relative gene expression was quantified and analyzed by the 2^−∆∆*C*t^ method.

### 4.8. Quantitative Analysis of GFP-mRFP-LC3 Dots

Panc-1 cells were transfection with GFP-mRFP-LC3 and reseeded into 96-well plates at a density of 4000 cells per well. After designated treatments, GFP-mRFP-LC3 fluorescence was observed using a confocal microscope LSM710 (Carl Zeiss, Oberkochen, Germany).

### 4.9. siRNA Transfection

Mia PaCa-2 and Panc-1 cells were grown in 6-well plates and transfected with 50 nM siRNA using Lipofectamine 2000 (Invitrogen) according to the manufacturer’s protocol. siRNA targeting Atg5 mRNA (AGUGAACAUCUGAGCUACCCGGAUA) and siRNA control duplexes were purchased from RiboBio company (Guangzhou, China).

### 4.10. Statistical Analysis

Results are expressed as the means ± SD unless stated otherwise. Statistical analysis was performed using the Student’s *t*-test, with a *p*-value < 0.05 considered statistically significant. All experiments were repeated at least three times. All analyses were conducted using GraphPad Prism 5 software (GraphPad Software Inc., La Jolla, CA, USA).

## 5. Conclusions

We found that inhibition of autophagy by deguelin markedly augmented doxorubicin-induced cell death in pancreatic cancer cells in vitro. Suppression of autophagy could be an effective novel strategy to overcome chemoresistance. Thus, deguelin could potentially be further developed as an important autophagy inhibitor to enhance anti-cancer efficacy of current DNA-damaging agents. Combined treatment with deguelin and doxorubicin represents an effective strategy for treating pancreatic cancer.

## Supplementary Materials

Supplementary materials can be found at www.mdpi.com/1422-0067/18/2/370/s1.
