# Supplementary material for: Inhibition of Autophagy by Deguelin Sensitizes Pancreatic Cancer Cells to Doxorubicin
Source: Int J Mol Sci. 2017 Feb 10;18(2):370. doi: 10.3390/ijms18020370 (PMC5343905; doi:10.3390/ijms18020370)
Supplement: Supplementary file 1 [file ijms-18-00370-s001.pdf]

Supplementary Materials: Inhibition of Autophagy by Deguelin Sensitizes Pancreatic Cancer Cells to Doxorubicin

Xiao Dong Xu, Yan Zhao, Min Zhang, Rui Zhi He, Xiu Hui Shi, Xing Jun Guo, Cheng Jian Shi, Feng Peng, Min Wang, Min Shen, Xin Wang, Xu Li and Ren Yi Qin

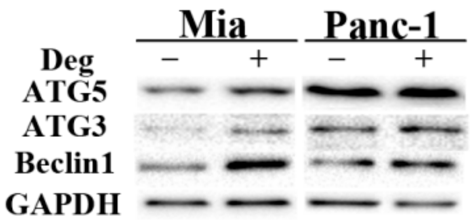

Figure S1. Levels of autophagy-related proteins increased after deguelin treatment. Western blot analysis of autophagy-related protein levels after Mia PaCa-2 and Panc-1 cells were treated with 25 μM deguelin for 24 h. Levels of Beclin1, Atg3, and Atg5 in cell extracts were analyzed by western blot using specific antibodies. GAPDH: glycerinaldehyde-3-phosphat-dehydrogenase

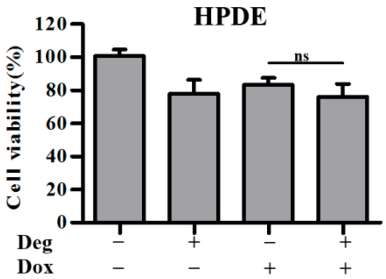

Figure S2. Deguelin does not enhance doxorubicin-induced cell death via suppression of autophagy in normal human pancreatic ductal (HPDE) cells. HPDE cells were treated with deguelin (25 μM), doxorubicin (2.5 μM), or deguelin plus doxorubicin for 24 h. Cell viability was analyzed by CCK-8 assay. Results are presented as the means ± SD. NS, the difference is not significant.

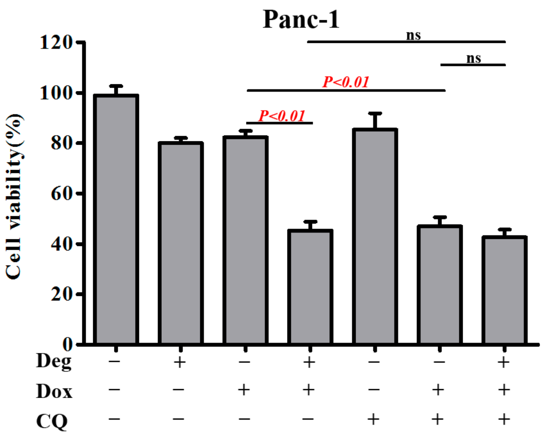

Figure S3. Deguelin enhances doxorubicin-induced cancer cell death via autophagy suppression. Panc-1 cells were treated with 2.5 μM doxorubicin alone or in combination with 25 μM deguelin or 10 μM chloroquine for 24 h. Cell viability was analyzed by CCK-8 assay. Results are presented as the means ± SD. NS, the difference is not significant.
